# Characterization of Early-Onset Finger Osteoarthritis-Like Condition Using Patient-Derived Induced Pluripotent Stem Cells

**DOI:** 10.3390/cells10020317

**Published:** 2021-02-04

**Authors:** Yeri Alice Rim, Yoojun Nam, Narae Park, Kijun Lee, Hyerin Jung, Seung Min Jung, Jennifer Lee, Ji Hyeon Ju

**Affiliations:** 1 Catholic iPSC Research Center, College of Medicine, The Catholic University of Korea, Seoul 06591, Korea; llyerill0114@gmail.com (Y.A.R.); givingtreemax@gmail.com (Y.N.); narae5322@catholic.ac.kr (N.P.); bluebusker@gmail.com (K.L.); ilovehyelin@catholic.ac.kr (H.J.); pobi28@naver.com (S.M.J.); 2 Department of Internal Medicine, Division of Rheumatology, Institute of Medical Science, College of Medicine, Seoul St. Mary’s Hospital, The Catholic University of Korea, Seoul 06591, Korea; poohish@naver.com

**Keywords:** chondrogenesis, human induced pluripotent stem cell, IL-6, MMP1, MMP10

## Abstract

Early osteoarthritis (OA)-like symptoms are difficult to study owing to the lack of disease samples and animal models. In this study, we generated induced pluripotent stem cell (iPSC) lines from a patient with a radiographic early-onset finger osteoarthritis (efOA)-like condition in the distal interphalangeal joint and her healthy sibling. We differentiated those cells with similar genetic backgrounds into chondrogenic pellets (CPs) to confirm efOA. CPs generated from efOA-hiPSCs (efOA-CPs) showed lower levels of *COL2A1*, which is a key marker of hyaline cartilage after complete differentiation, for 21 days. Increase in pellet size and vacuole-like morphologies within the pellets were observed in the efOA-CPs. To analyze the changes occurred during the development of vacuole-like morphology and the increase in pellet size in efOA-CPs, we analyzed the expression of OA-related markers on day 7 of differentiation and showed an increase in the levels of *COL1A1*, *RUNX2*, *VEGFA*, and *AQP1* in efOA-CPs. IL-6, MMP1, and MMP10 levels were also increased in the efOA-CPs. Taken together, we present proof-of-concept regarding disease modeling of a unique patient who showed OA-like symptoms.

## 1. Introduction

Osteoarthritis (OA) is a common form of arthritis that affects a significant portion of the elderly population worldwide. The cartilage that covers the end of bones is damaged or worn out in OA. OA can affect any joint in the human body; however, hands, knees, or hips are most commonly affected by the disease. Hand OA usually affects the carpometacarpal joint and interphalangeal joint and occurs most commonly in females over 50 years of age [1,2]. 

Although the negative effect of hand OA on quality of life is well known, data regarding hand OA development and pathogenesis remain limited, owing to the lack of samples and animal models [3]. While in vitro disease modeling is used as a tool to study the mechanisms of cartilage-related diseases, primary chondrocytes are difficult to obtain, and they easily lose their phenotype and characteristics under culture conditions. These challenges can now be overcome using human induced pluripotent stem cells (hiPSCs), a breakthrough in experimental modeling of human diseases [4]. The development of hiPSCs has also opened new avenues in tissue engineering and regenerative medicine. Induction of the expression of transcription factors such as *OCT4*, *SOX2*, *KLF4*, and *MYC* converts somatic cells into a state that is similar to embryonic stem cells [5,6]. Modeling of cartilage and cartilage-related diseases such as rheumatoid arthritis and knee OA have been studied using hiPSCs [1,7,8]. Castro-Viñuelas et al. have generated and characterized hiPSCs from fibroblasts derived from a patient with hand OA [1]. Two cell lines were generated from 52- and 76-year-old female patients. The cells obtained after chondrogenic differentiation of these cell lines showed lower levels of collagen and proteoglycan than those of the healthy controls. However, owing to the lack of data on unique OA-like conditions, further investigations are required for improving our understanding regarding these conditions, as well as of OA.

In this study, we aimed to develop and characterize an in vitro model of early onset finger OA (efOA) from a patient with radiographic efOA-like condition in the distal interphalangeal joint using patient-derived hiPSCs. We selected this patient for further analysis since only cartilage destruction and joint space narrowing were observed in the distal interphalangeal joints of all fingers, without any signs of inflammation or autoimmunity, which was interesting. We obtained the dermal fibroblasts from the skin biopsy specimen of the patient and generated hiPSCs. The patient had one sibling of similar age with no symptoms of cartilage destruction. hiPSCs generated similarly from her healthy sibling was used as the negative control. Using these two cell lines, we generated chondrogenic pellets (CPs) to confirm the unique characteristics of the patient. This study presents proof-of-concept regarding modeling of early onset OA-like disease using patient-derived hiPSCs.

## 2. Materials and Methods

### 2.1. Patient Consent and Ethical Procedures

The study was approved by the Institutional Review Board/Ethics Committee of the Catholic University of Korea, Seoul, Korea (approval number: KC19SNSI0819). Written informed consent was obtained from each participant.

### 2.2. Dermal Fibroblast Isolation and Maintenance

Dermal fibroblasts were isolated using the method of Rim et al. (2018) [5]. Skin samples were obtained via skin punch biopsy procedures from individuals, both of whom were females. Chopped and homogenized skin tissue was resuspended in Dulbecco’s modified Eagle’s medium (DMEM, Gibco, Carlsbad, CA, USA) containing 0.01% collagenase. The tissues were digested for 4 h at 37 °C with vigorous shaking. The cells were then washed and resuspended in DMEM supplemented with 20% fetal bovine serum (FBS, Gibco) and 1% penicillin/streptomycin solution (Gibco). The isolated cells were cultured until the cells reached more than 80% confluence.

### 2.3. hiPSC Generation Using Patient-Derived Dermal Fibroblasts

hiPSCs were generated using the method of Ju et al. [5]. The cells were detached with trypsin/EDTA and 2 × 10^5^ cells were obtained per well. The cells were resuspended in 20% DMEM and seeded into a 6-well plate. The next day, pre-aliquoted Sendai virus (Thermo Fisher Scientific, Carlsbad, CA, USA) was added to the cells and incubated in the presence of 5% CO_2_ at 37 °C for 48 h. The virus-containing media was removed after 48 h. Cell media was changed every other day for six days. On day 7, the media was changed to Essential 8 (E8, Thermo Fisher Scientific) media and cultured until colonies appeared. The media was changed daily after it transitioned to E8 media. Three clones of iPSCs were generated from each individual.

### 2.4. Chondrogenic Differentiation Using Pellet Culture

Chondrogenic differentiation was conducted using our previous methods [5,9,10,11,12]. A 1:1 mixture of E8 media (Thermo Fisher Scientific) and Aggrewell media (StemCell Technologies, Vancouver, BC, Canada) was added to hiPSCs (1–5 × 10^4^ cells per cm^2^) for embryonic body (EB) formation, which were maintained in a petri dish with E8 media and 10 µM rho-associated kinase (ROCK) inhibitor (ROCKi) for 24 h. The formed EB clusters were then maintained in E8 media without ROCKi for an additional 48 h. The culture media was then transferred to EB maintenance media containing Dulbecco’s Modified Eagle Medium/Nutrient Mixture F-12 (DMEM/F-12, Thermo Fisher Scientific), 7.5% NaHCO_3_ (Thermo Fisher Scientific), 64 ng/mL ascorbic acid 2-phosphate (Sigma Aldrich, St. Louis, MO, USA), 14 ng/mL sodium selenite (Sigma Aldrich), 10.7 ng/mL transferrin (Sigma Aldrich), 20 ng/mL insulin (Thermo Fisher Scientific), and 2 ng/mL TGFβ1 (Peprotech, Rocky Hill, NJ, USA), and cultured for an additional 72 h. Outgrowth (OG) cells are used for chondrogenic pellet formation. To induce OG cells, the EBs were harvested and 50–70 EB per cm^2^ were seeded in a gelatin-coated culture dish with OG induction media consisting of DMEM (Thermo Fisher Scientific), 20% FBS (Thermo Fisher Scientific), and 10% penicillin/streptomycin (Thermo Fisher Scientific) supplemented with ROCKi for attachment. OGF cells were induced from the attached EBs for 72 h in the presence of 5% CO_2_ at 37 °C. Next, the remaining EBs and the induced OG cells was detached with trypsin/EDTA (Thermo Fisher Scientific) and harvested as single cells. The harvested OG cells were plated onto a new gelatin-coated dish (1–5 × 10^4^ cells per cm^2^). For OA characterization, only passage 1 OG cells were used. The cells were harvested and distributed in a 15 mL conical tube (3 × 10^5^ cells per tube) and the medium was changed to chondrogenic differentiation media (CDM; DMEM supplemented with 20% knockout serum replacement, 1× non-essential amino acids, 1 mM l-glutamine, 1% sodium pyruvate, 1% Insulin-Transferrin-Selenium (ITS)+ Premix, 10^−7^ M dexamethasone, 50 mM ascorbic acid, and 40 μg/mL l-proline) supplemented with 10 ng/mL recombinant human TGF-β3. The cells resuspended in CDM were centrifuged at 750× *g* for 5 min. The pellets obtained were maintained for 21 days and the media was changed every three days.

### 2.5. Real Time-Polymerase Chain Reaction (PCR)

CPs or cells were harvested and stored at −80 °C after snap freezing in liquid nitrogen and grinding using a pestle. The ground samples were incubated with TRIzol (Thermo Fisher Scientific) and mRNA was extracted according to the manufacturer’s instructions. A RevertAid first strand cDNA synthesis kit (Thermo Fisher Scientific) was used for cDNA synthesis. Approximately 2 μg of the extracted total RNA was used to synthesize cDNAs. Real-time PCR was performed using a LightCycler^®^ 480 Instrument II (Roche, Basel, Switzerland). *GAPDH* was used as an internal control for normalization of gene expression. The primer sequences are listed in Table 1.

### 2.6. Immunocytochemical Staining of iPSCs

To achieve hiPSC colonies, 2 × 10^3^ iPSCs were seeded in one well of a vitronectin-coated 6-well plate for each staining. Cells were expanded for 5–7 days with E8 media which was changed daily. The staining process started with a wash with PBS and cells were fixed with 4% paraformaldehyde (Biosesang, Seongnam, Republic of Korea). After another wash with PBS, cells were permeabilized using 0.1% triton X-100 (Biosesang) for 10 min, then blocked for 30 min at room temperature (RT) with PBS supplemented with 2% bovine serum albumin (BSA; Sigma-Aldrich, St. Louis, MO, USA) (PBA). Primary antibodies were diluted in PBA and incubated for 2 h at RT. The following dilution factors were used in this experiment: SSEA4 (1:200; EMD Millipore, Billerica, MA, USA), TRA-1-60 (1:100; EMD Millipore), TRA-1-81 (1:100; EMD Millipore), OCT4 (1:100; Santa Cruz Biotechnology, Dallas, TX, USA), SOX2 (1:100; BioLegend, San Diego, CA, USA), KLF4 (1:250; Abcam, Cambridge, UK). After washing the cells, Alexa Fluor 594-(1:400; Life Technologies, Carlsbad, CA, USA) and 488-(1:400; Life Technologies) conjugated secondary antibodies were diluted in PBA and incubated for 1 h eat RT avoiding light. Cells were then washed and mounted using ProLong Antifade mounting reagent (Thermo Fisher Scientific). Colonies were detected under an immunofluorescence microscope.

### 2.7. Histological Analysis of Pellets

The method of Ju et al. (2018) was used for histological analysis [5]. The CPs were washed with phosphate-buffered saline (PBS) and fixed in 4% paraformaldehyde for 2 h RT. The samples were dehydrated using an ascending series of ethanol (Biosesang) solutions. Additional clearing was performed with sequential ethanol-xylene mixtures, and the pellets were infiltrated overnight with paraffin. Paraffin blocks were fixed and 7 μm sections were obtained using a microtome. Before staining the sections, the slides were placed in a 60 °C oven for at least 10 min. Following this, the slides were immediately deparaffinized using xylene, rehydrated sequentially using a descending series of ethanol solutions, and rinsed with running tap water for 1 min each. Next, the sections were incubated in 1% Alcian blue solution for 30 min, following which the slides were washed and counterstained with Nuclear fast red for 1 min. The slides were washed in running tap water and dried for 10 min until complete dryness. After the staining process, the slides were dehydrated sequentially using an ascending series of ethanol. Ethanol was cleared with two cycles of 100% xylene and the slides were mounted with VectaMount permanent mounting medium (Vector Laboratories, Burlingame, CA, USA).

### 2.8. Inflammatory Cytokine and Matrix Metalloproteinase (MMP) Array

Both the inflammatory cytokine array and MMP array kits were purchased from Abcam. The process was performed according to the manufacturer’s instructions. Each membrane with the specific antibody array was placed in a tray. The membranes were blocked with blocking buffer at RT for 30 min. The cultured media supernatant of each group was added to the membranes and incubated for 2 h at RT. After several washes, the membranes were incubated with biotin-conjugated anti-cytokine antibodies for 2 h at RT. After another wash, horse radish peroxidase-conjugated streptavidin was added to the membranes and incubated for 2 h at RT. The membranes were washed again and placed on a plastic sheet for visualization. A mixture of detection buffer was added to each membrane and incubated for 2 min. Another plastic sheet was placed on top of the membrane, excess buffer was removed, and the signals were assessed using a Fusion SL imaging system (Vilber Lourmat, Marne-la-Vallee, France).

### 2.9. Statistical Analysis

All experiments were repeated three or more times using three clones of each cell line derived from the individual. Five CPs were generated from each hiPSC clone and were used for each trial. The data are presented as mean ± standard deviation. Statistical analysis was performed, and graphs were generated using GraphPad Prism 5 (GraphPad). A *t*-test was used to analyze non-parametric quantitative datasets and one-tailed *p*-values were calculated. Here, statistical significance is indicated as follows: * *p* < 0.05; ** *p* < 0.01; *** *p* < 0.001. One-way ANOVA, followed by Dunnett’s post-hoc test was used to analyze the IL-1β treatment experiment and is indicated as follows: # *p* < 0.05; ## *p* < 0.01; ### *p* < 0.001.

## 3. Results

### 3.1. Generation of hiPSCs from Fibroblasts of a Patient with Early-Onset Finger OA (efOA) 

**Figure 1:**
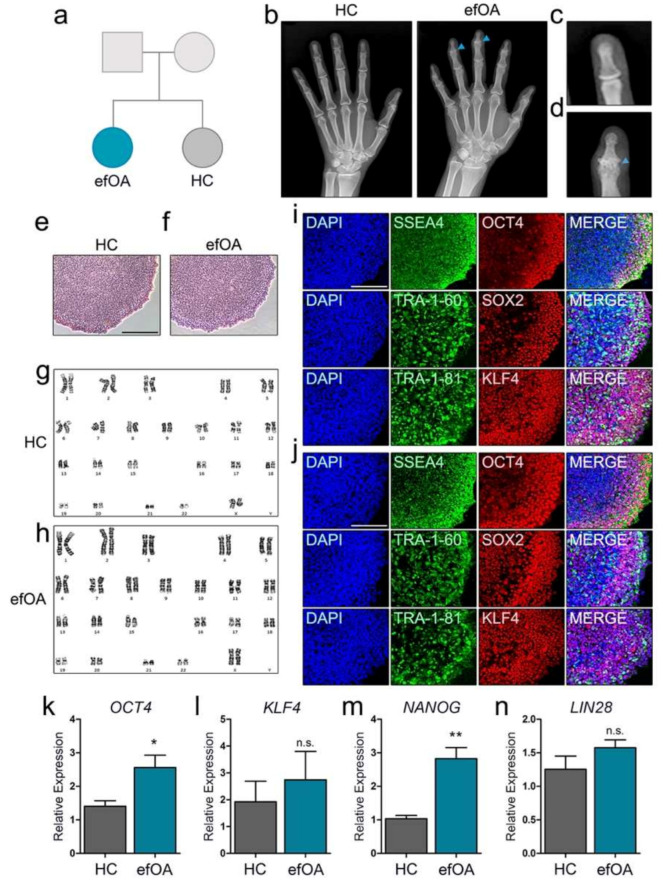
Generation of patient-derived human induced pluripotent stem cells (hiPSCs). (**a**) The family tree of the patient with early finger osteoarthritis (efOA) and her sibling (HC). (**b**) Radiographic image of the hands of the HC and the patient. (**c**) Radiographic image of the distal interphalangeal joint of HC. (**d**) Radiographic image of the distal interphalangeal joint of the patient. (**e**) Bright field image of alkaline phosphatase-stained HC-hiPSCs and (**f**) efOA-hiPSCs. (**g**) Normal karyotype of HC-hiPSCs. (**h**) Normal karyotype of efOA-iPSCs. (**i**) Immunofluorescence staining with pluripotent markers in HC-hiPSCs. (**j**) Immunofluorescence staining with pluripotent markers in efOA-hiPSCs. All scale bars represent 200 μm. (**k**) Relative expression of *OCT4*. (**l**) Relative expression of *KLF4*. (**m**) Relative expression of *NANOG*. (**n**) Relative expression of *LIN28*. (* *p* < 0.05, ** *p* < 0.01).

Dermal fibroblasts were isolated from the skin biopsy samples of the patient (efOA) and sibling (HC) (Figure 1a). The patient with efOA exhibited a radiographic erosive finger OA-like morphology in the distal interphalangeal joints at a relatively early age, whereas the HC sibling did not show any symptoms of this type (both in their early 30s) (Figure 1b). No OA-like symptoms were detected in the knee or hip in either individual. In addition, no signs of inflammation or autoimmunity was evident in the blood tests of the patient, which led to the diagnosis of early onset OA. The distal interphalangeal joint of the patient (Figure 1d) showed the thinning of the cartilage due to severe degradation. Bone spur generation induced by the significantly reduced joint space between the distal phalanx and the middle phalanx was also observed in the patient (Figure 1c). Cell lines of hiPSCs were generated from the isolated fibroblasts using a Sendai RNA virus to minimize the risk of genomic abnormalities. The newly formed iPSC colonies were obtained from the transduced fibroblasts and expanded to generate various cell lines from each individual. The generated hiPSC lines were positively stained for alkaline phosphatase, which is a key marker of pluripotency in embryonic stem cells (Figure 1e,f). All cell lines exhibited normal karyotype (Figure 1g,h). The pluripotency of the generated hiPSCs was confirmed using additional pluripotency markers such as SSEA4, TRA-1-60, TRA-1-81, OCT4, SOX2, and KLF4. Both HC-hiPSCs (Figure 1i) and efOA-hiPSCs (Figure 1j) showed positive expression of pluripotent markers. The relative expression of *OCT4*, *KLF4*, *NANOG*, and *LIN28* in the generated hiPSCs was assessed using real-time PCR. *OCT4* and *NANOG* expression was higher in the efOA-hiPSCs compared to that in the control (Figure 1k,m), whereas *KLF4* and *LIN28* levels did not differ significantly between the hiPSCs derived from the patients and the control (Figure 1l,n). Taken together, we successfully generated hiPSCs from HC- and efOA-derived dermal fibroblasts and confirmed that the generated hiPSC cell lines were pluripotent.

### 3.2. Chondrogenic Differentiation Using efOA-hiPSCs

**Figure 2:**
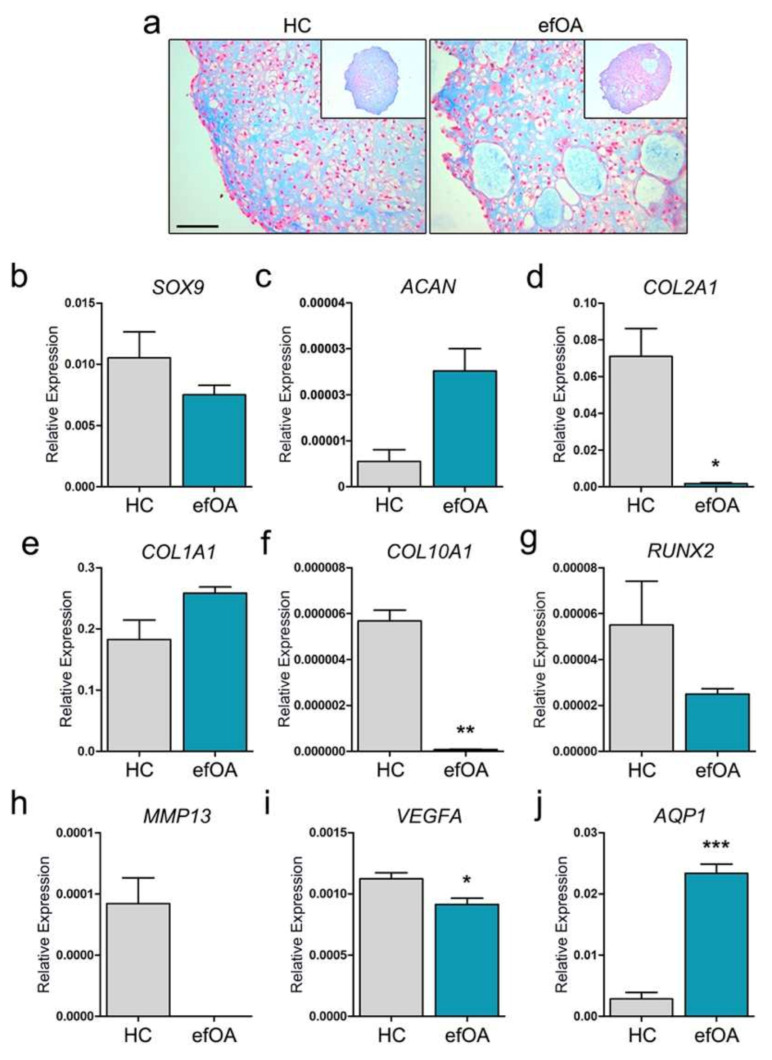
Characterization of completely differentiated chondrogenic pellets (CPs) on day 21. (**a**) Alcian blue staining of day 21 CPs. Scale bar represents 100 μm. (**b**) Relative expression of *SOX9*. (**c**) Relative expression of *ACAN*. (**d**) Relative expression of *COL2A1*. (**e**) Relative expression of *COL1A1*. (**f**) Relative expression of *COL10A1*. (**g**) Relative expression of *RUNX2*. (**h**) Relative expression of *MMP13*. (**i**) Relative expression of *VEGFA*. (**j**) Relative expression of *AQP1*. The expression of each gene was normalized to that of *GAPDH*. (* *p* < 0.05, ** *p* < 0.01, *** *p* < 0.001).

Cartilage destruction is the primary hallmark of all types of OA-like conditions. To confirm the quality of the cartilage of the patient indirectly, we completely differentiated efOA-hiPSCs into CPs for 21 days. Interestingly, the CPs derived from efOA-hiPSCs (efOA-CPs) were large and contained vacuole-like morphologies within the pellets (Figure 2a). There is a decreasing trend of SOX9 expression in efOA-CPs that was not significant (Figure 2b). Aggrecan (*ACAN*) and Type II collagen (*COL2A1*) are the two major proteins in hyaline cartilage. *ACAN* expression was higher in efOA-CPs; however, there was no significance due to the low expression in both groups (Figure 2c). We confirmed that *COL2A1* expression was significantly low in the completely differentiated efOA-CPs (Figure 2d). *COL1A1*, which is a fibrotic cartilage marker, as well as a hypertrophic marker, was similarly expressed in both HC- and efOA-CPs (Figure 2e). We hypothesized that the level of the hypertrophic cartilage marker, *COL10A1*, might be high in efOA-CPs, which might induce vacuole-like characteristics, and observed that *COL10A1* level was significantly low in the 21-day-old efOA-CPs (Figure 2f). The expression of other hypertrophic markers was also confirmed in the efOA-CPs. The level of *RUNX2*, which is the main regulator of cartilage hypertrophy, did not differ significantly between the two groups (Figure 2g). The expression of the hypertrophy markers *MMP13* and vascular endothelial growth factor A (*VEGFA*) were low in efOA-CPs (Figure 2h,i). In the case of *MMP13*, day 21 efOA-CPs showed no expression in every trial. The use of AQP1 as a marker for OA, as suggested in several previous studies, remains controversial [13,14]. We observed that *AQP1* expression in efOA-CPs increased significantly after chondrogenic differentiation (Figure 2j). Taken together, we confirmed that the characteristics of efOA-CPs differed from those of HC-CPs after 21 days of differentiation.

### 3.3. Characterization of efOA Using Early-Stage CPs

**Figure 3:**
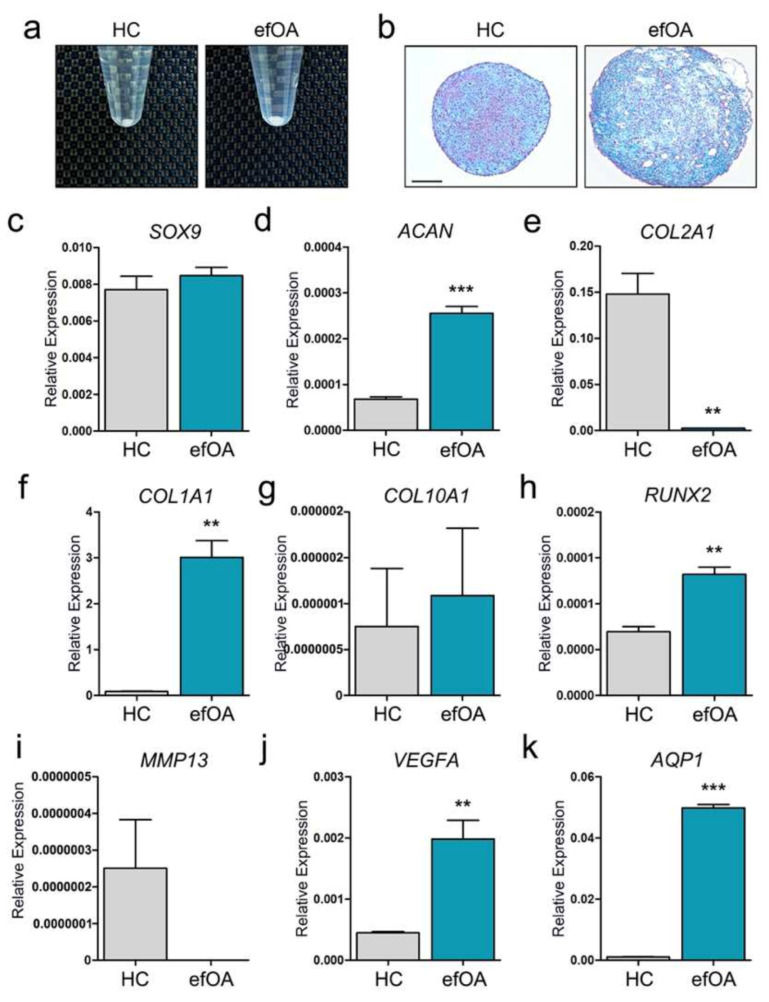
Characterization of day 7 early stage chondrogenic pellets (CPs). (**a**) Image of day 7 CPs. (**b**) Alcian blue staining of day 7 CPs. Scale bar represents 200 μm. (**c**) Relative expression of *SOX9*. (**d**) Relative expression of *ACAN*. (**e**) Relative expression of *COL2A1*. (**f**) Relative expression of *COL1A1*. (**g**) Relative expression of *COL10A1*. (**h**) Relative expression of *RUNX2*. (**i**) Relative expression of *MMP13*. (**j**) Relative expression of *VEGFA*. (**k**) Relative expression of *AQP1*. The expression of each gene was normalized to that of *GAPDH*. (** *p* < 0.01, *** *p* < 0.001).

While we have characterized and analyzed the completely differentiated day 21 CPs, it was important to determine the cause of the vacuole-like morphologies inside the efOA-CPs. The difference in pellet size first appeared during the early phase (approximately days 5 to 7) of the differentiation process (Figure 3a). Vacuole-like structures were also found in day 7 efOA-CPs; however, the size was relatively smaller than that of day 21 efOA-CPs (Figure 3b). Therefore, we decided to analyze the expression of chondrogenic and hypertrophic markers in differentiated CP on day 7. *SOX9* expression did not differ significantly between the two groups, even on day 7 (Figure 3c). Interestingly, the expression of *ACAN* was significantly higher in day 7 efA-CPs (Figure 3d). *COL2A1* expression was similar to that in day 21 CPs and decreased significantly on day 7 (Figure 3e). Unlike the expression on day 21, *COL1A1* was highly expressed in efOA-CPs on day 7 (Figure 3f). However, *COL10A1* expression did not differ between the two groups (Figure 3g). To confirm the reliability of the results in Figure 3, the expression levels of chondrogenic and hypertrophic markers were further analyzed using two more reference genes (Figure S1). Based on previous studies, reference genes, *β-actin* (*ACTB*) and *18S RNA* were selected for analysis additional to *GAPDH* [15,16]. While the expression calculated using *18S RNA* showed a relatively lower levels, we confirmed similar significances between the results calculated using three reference genes.

Successful chondrogenesis using hiPSCs is critical to compare the difference between HC and efOA-CP characteristics. To confirm whether the chondrogenic differentiation process was successful during this study, the expression of chondrogenic markers in day 7 and 21 CPs were compared to that of the hiPSCs of each group as a control (Figure S2). We first confirmed the disappearance of the hiPSC marker, *LIN28*, in the CPs of each group (Figure S2a). *SOX9*, a transcription factor critical for chondrogenesis, was significantly increased as the hiPSCs were differentiated into CPs (Figure S2b). *ACAN* was also significantly increased in the CPs, while both groups showed the highest expression on day 7 of differentiation (Figure S2c). *COL2A1* also showed a similar expression pattern to that of *ACAN* (Figure S2d). The expression of *COL1A1* was significantly increased in the CPs of each group compared to the hiPSCs (Figure S2e). While the expression of *COL1A1* increased gradually in the HC group, peaking on day 7 of differentiation in the efOA group. Interestingly, *COL10A1* was not expressed in either hiPSCs (Figure S2f). Hypertrophic markers were analyzed in the day 7 pellets as well. While *RUNX2* expression did not differ significantly between the two cell lines on day 21, its expression increased significantly in efOA-CPs on day 7 (Figure 3h). Similar to the results of day 21 efOA-CPs, *MMP13* expression was not shown on day 7 efOA-CPs (Figure 3i). *VEGFA* expression increased significantly in efOA-CPs on day 7 compared to that in day 21 CPs (Figure 3j). Interestingly, *AQP1* was also significantly expressed on day 7 (Figure 3k). Taken together, we confirmed a significant increase in the expression of hypertrophic markers in the efOA-CPs on day 7, which might be associated with the significant changes in the size and internal structure of the efOA-CPs.

We further compared several characteristics in day 7 and 21 CPs. The size of HC-CPs was similar on day 7 and 21 (Figure S3a). The pellet size of efOA-CPs was increased on day 7 and further increased further until day 21 of differentiation. The size of efOA pellets at both time points was significantly larger than that of HC-CPs. The vacuole-like structures (vacuoles with noticeably larger size compared to lacunae structures) within the CPs were counted and the size of the vacuoles were measured. Day 7 HC-CPs did not show any formation of vacuole-like morphologies; however, efOA-CPs had a significantly increased number of vacuoles in their structure on day 7 of differentiation (Figure S3b) Vacuoles were also found in day 21 HC-CPs, and the higher count of vacuoles was maintained in day 21 efOA-CPs. The size of vacuoles increased on day 21 of differentiation in both groups, and efOA-CPs showed a significantly larger size of vacuole on day 21 (Figure S3c). We also observed that the vacuole size in day 21 efOA-CPs was significantly larger than that of vacuoles within the day 21 HC-CPs. These results confirm that the significant changes in structure found in efOA-CPs appeared during a relatively early stage of differentiation. We assumed that the increased size of efOA-CPs might be caused by the expanding size of vacuoles within the pellets.

### 3.4. Cytokine and MMP Analysis in Early Stage efOA-CP Culture Media Supernatant

**Figure 4:**
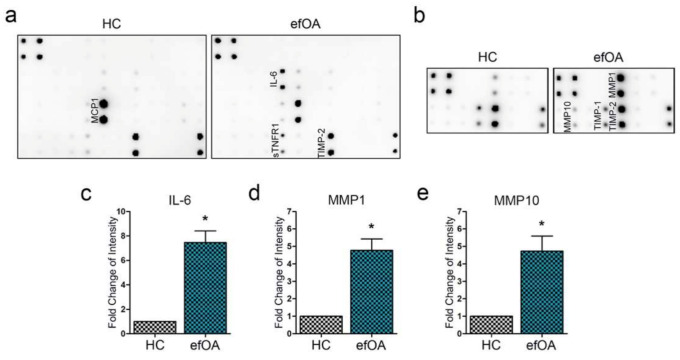
Inflammatory cytokine and MMP array using conditioned supernatant of day 7 early stage chondrogenic pellets (CPs). (**a**) Results of inflammatory cytokine array of HC- and efOA-CP culture supernatant. (**b**) Results of the MMP array of HC- and efOA-CP culture supernatant. (**c**) Fold change levels of measured intensity of IL-6, (**d**) MMP1, and (**e**) MMP10. (* *p* < 0.05).

Early stage efOA-CPs showed initiation of increase in pellet size and formation of vacuole-like structures within the pellet. To identify the candidate proteins that might induce the above characteristics of efOA-CPs and are related to the increased expression of hypertrophic markers, we used arrays against inflammatory cytokines and MMPs. We observed that interleukin (IL)-6 level was high in the culture supernatant of the efOA-CPs on day 7 (Figure 4a). TIMP-1 and TIMP-2 were expressed in both groups, which are reported to be secreted in chondrocytes. Both HC- and efOA-CPs secreted similar levels of MCP1, sTNFR1, and TIMP-2. MMP1 and MMP10 expression increased in the culture supernatant of efOA-CPs (Figure 4b), with the level of MMP1 being higher than that of MMP10. The IL-6 and MMP1 levels were significantly higher in early stage efOA-CP supernatant than in the HC-CP supernatant (Figure 4c–e). Thus, we confirmed that IL-6, MMP1, and MMP10 expression increased significantly in the culture supernatant of early stage (day 7) efOA-CPs.

### 3.5. Analysis of Inflammatory Cytokines and MMPs in Early and Late Stage efOA-CPs

**Figure 5:**
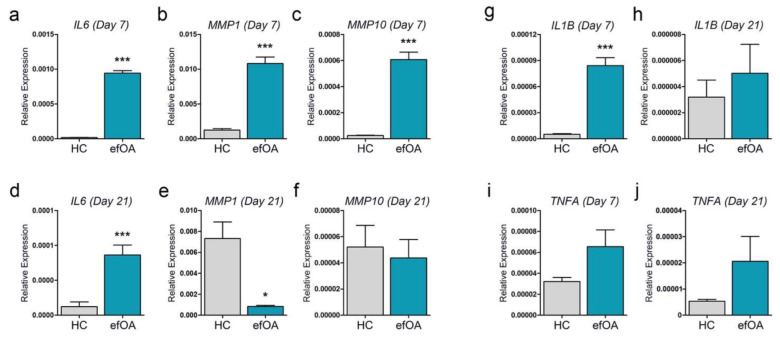
Analysis of selected inflammatory cytokines and MMPs using real time-PCR. (**a**) Relative expression of *IL6* on day 7. (**b**) Relative expression of *MMP1* on day 7. (**c**) Relative expression of *MMP10* on day 7. (**d**) Relative expression of *IL6* on day 21. (**e**) Relative expression of *MMP1* on day 21. (**f**) Relative expression of *MMP10* on day 21. (**g**) Relative expression of *IL1B* on day 7. (**h**) Relative expression of *IL1B* on day 21. (**i**) Relative expression of *TNFA* on day 7. (**j**) Relative expression of *TNFA* on day 21. The expression of each gene was normalized to that of *GAPDH*. (* *p* < 0.05, *** *p* < 0.001).

To confirm the results shown in Figure 4, we performed real-time PCR for *IL6*, *MMP1*, and *MMP10*. *IL6* expression increased significantly in efOA-CPs on day 7, confirming the result shown in Figure 4a (Figure 5a). *MMP1* and *MMP10* expression increased significantly in day-7-old efOA-CPs (Figure 5b,c). We confirmed the expression of the specific markers selected using day 7 pellets in completely differentiated day 21 CPs. Although generally low, *IL6* expression in the 21-day-old efOA-CPs exceeded that in the HC, similar to that observed on day 7 (Figure 5d). In contrast, the high expression of *MMP1* on day 7 was significantly reversed on day 21 in (Figure 5e). In addition, the expression of *MMP10* on day 21 was lower than that on day 7, although the expression levels were similar between the two groups (Figure 5f). IL-1β and tumor necrosis factor (TNF)α are important pathological candidates related to OA development, which have also been reported to increase IL-6 expression [17,18,19]. Therefore, we decided to confirm the expression of these two representative cytokines in CPs on day 7 and day 21. Interestingly, *IL1B* expression increased significantly in efOA-CPs on day 7 (Figure 5g); however, the increased levels disappeared in the completely differentiated efOA-CPs (Figure 5h). *TNFA* expression appeared to increase in efOA-CPs at both time points; however, no significant difference was observed between the two groups (Figure 5i,j). The results in Figure 5 were also confirmed using two additional reference genes namely β-actin (*ACTB*) and *18S RNA* (Figure S4). The results calculated with the two additional housekeeping genes showed lower significance than that of *GAPDH*; however, the patterns of significance were mostly similar. Based on these results, we confirmed that *IL6*, *MMP1*, and *MMP10* expression corresponded to their protein levels in the culture supernatant, and that IL-1β expression might be associated with these changes.

### 3.6. Analysis of Inflammatory Cytokines and MMPs in Early and Late Stage efOA-CPs

**Figure 6:**
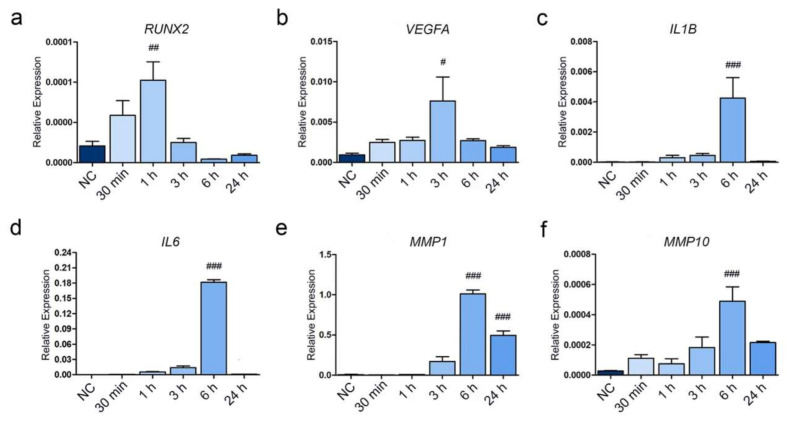
Confirmation of gene expression in IL-1β treated CPs. (**a**) Relative expression of *RUNX2*. (**b**) Relative expression of *VEGFA*. (**c**) Relative expression of *IL1B*. (**d**) Relative expression of *IL6*. (**e**) Relative expression of *MMP1*. (**f**) Relative expression of *MMP10*. Statistically significant differences between the normal control (NC) and the treatment groups are indicated by the hash symbol (One-way ANOVA, Dunnett’s test, # *p* < 0.05, ## *p* < 0.01, ### *p* < 0.001).

IL-1β is associated with inflammation in OA, the level of which increased on day 7 in efOA-CPs in this study. To confirm whether IL-1β is associated with the increased expression of IL-6 which was followed by the increased levels of MMP1 and MMP10, we treated HC-CPs with IL-1β and harvested samples at different time points for analysis. Expression of *RUNX2*, the transcription factor responsible for hypertrophic differentiation, increased and peaked after 1 h of IL-1β treatment (Figure 6a). *VEGFA* expression also started increasing significantly from 30 min post-treatment, and the expression was stably maintained for 24 h (Figure 6b). Treatment with IL-1β also increased the expression levels of *IL1B*, and the expression peaked after 6 h of treatment (Figure 6c). To confirm the effect of IL-1β on the expression of *IL6*, *MMP1*, and *MMP10*, the levels of the three selected markers were assessed. *IL6* expression started to increase 30 min after IL-1β treatment and sharply increased 6 h after treatment (Figure 6d). *MMP1* expression increased considerably after IL-1β treatment. *MMP1* expression was highest after 6 h of treatment and decreased after 24 h; however, high level of *MMP1* was still maintained (Figure 6e). *MMP10* level also increased after IL-1β treatment; however, the levels were not as high as that of *MMP1* (Figure 6f). The expression levels of *IL6*, *MMP1*, and *MMP10* were further analyzed using two more housekeeping genes, namely *ACTB* and *18S RNA* to confirm our result (Figure S5). While the expression levels varied, we confirmed similar significances. These results suggest that the confirmed target genes, *IL6*, *MMP1*, and *MMP10*, might be associated to IL-1β, which is an inflammatory cytokine that plays a critical role in OA.

## 4. Discussion

Chondrocytes and cartilage tissues in OA (especially in efOA) are difficult to study due to the lack of clinical tissue samples. The procedure to obtain these samples is invasive, which renders healing difficult, as cartilage tissues cannot regenerate by themselves. Hence, the generation of hiPSCs has been a breakthrough for studying these types of tissues.

Differentiated tissues or organoids generated from hiPSCs are efficiently used for tissue regeneration, drug screening, and disease modeling. Confirming the etiology and pathophysiology of the disease is critical for accomplishing these goals. Toward this, the development of a physiologically relevant experimental model of the disease is required [20], which is usually achieved via in vivo animal modeling; however, generation of animal models that can recapitulate the pathophysiology is difficult for certain diseases [21]. In addition, some experimental animal models cannot translate the biological responses of drugs to humans, which eventually leads to high failure rates in basic research and pharmaceutical research and development [20]. Therefore, an appropriate in vitro human disease model that can reflect the pathophysiological mechanism of the disease is strongly required. Although human primary cells are efficient candidates for disease modeling, their limited expansion rate renders them insufficient as sources of research raw materials. The unlimited expansion rate of patient-derived hiPSCs can overcome these shortcomings.

In the present study, we successfully generated hiPSCs from HC- and efOA-derived dermal fibroblasts and confirmed the different characteristics of CPs derived from both cell lines. We confirmed that IL-6, MMP1, and MMP10 levels were significantly increased in the culture supernatant of early-stage day 7 CPs; however, these significant changes disappeared in the completely differentiated CPs. We also suggest that these significant changes might be related to the increased expression of IL-1β.

CPs derived from efOA-hiPSCs showed morphological changes during days 3 to 7 of differentiation. Day 7 CPs showed significant increase in size, which we believe might be related to the generation of vacuole-like morphologies within the pellets (Figure 3a). Based on these observations, day 21 CPs were suspected to be too old to detect the changes between the two groups. Therefore, we analyzed the expression of chondrogenic and hypertrophic markers in day 7 differentiated CP. While aggrecan is usually known as a marker for hyaline cartilage, increased levels of aggrecan in synovial fluid was also confirmed as a marker for OA and ongoing cartilage destruction [22]. Interestingly, the expression of *ACAN* was significantly increased in day 7 efOA-CPs (Figure 3d). *SOX9* expression did not differ significantly between the two groups, even on day 7. At both time points, *COL2A1* expression decreased significantly in the efOA-CPs. *AQP1* expression increased significantly at both time points in the efOA-CPs. While significant changes in hypertrophic markers were not observed in day 21 CPs, *RUNX2* and *VEGFA* levels increased in day 7 CPs. The selection of the time point for analysis is critical to identify disease-relevant genes for disease modeling [23]. Although further analysis is required, we believe that the identification of these differences, along with determination of the ideal time point, might be critical for disease modeling using differentiated cells derived from hiPSCs.

The relationship between IL-6 and OA or cartilage destruction has been reported in several studies. IL-6 produced by a mixed population of inflammatory cells, such as macrophages, lymphocytes, and synoviocytes, is detected in early OA [24,25,26]. The concentration of IL-6 in synovial fluid correlated with radiographic OA severity [27]. A previous study confirmed that the levels of circulating IL-6 correlated with the development of radiographic knee OA [27]. Blumenfeld et al. found that single nucleotide polymorphisms (SNPs) in *IL6* were associated with the radiographic levels of hand OA, suggesting that specific DNA motifs in *IL6* might contribute to the development of hand OA [28,29]. In addition, in vitro and in vivo studies have reported that IL-6 enhances cartilage degradation [30]. Exogenous IL-6 promoted calcium-containing crystal formation and upregulated the genes involved in calcification, such as Ank, Annexin5, and Pit-1 in chondrocytes. The formation of calcium phosphate crystals stimulated IL-6 secretion in chondrocytes, indicating the existence of an autocrine loop [31].

MMP1 plays a critical role in cartilage degradation by affecting aggrecans and collagens in the cartilage tissue [32,33]. MMP1 levels in OA patient serum were significantly higher than that in healthy controls [34]. Both IL-6 and IL-1β treatment in meniscus cells increased the expression of MMP1 [35]. While the levels of IL-6 correlated with the severity of OA, it also correlated with the expression of MMP1 in OA synovial fluid [27]. Our results show a possibility that IL-6 induces MMP1 expression in efOA-CPs, which was consistent with the results of previous studies. IL-6 was previously reported to increase the expression of MMP1 in human primary monocytes via c-Jun upregulation and ERK and JNK cascades [36].

Our study has several limitations. While an increase in IL-6, MMP1, and MMP10 levels was observed, further confirmation via knockdown of IL-1β or IL-6 using siRNAs might be useful to validate our theory. The comparison of early onset OA iPSCs and late (progressive) OA iPSCs might also help us understand and compare these two types of diseases.

CTX-II, a biological marker, can reflect OA progression [37]. It is usually detected in serum, urine, and synovial fluid [38]. Collagen type II can be degraded by MMP1 into small fragments which are further degraded by other MMPs into CTX-II [39]. The measurement of OA and cartilage destruction related proteins such as CTX-II or aggrecan in the conditioned media might be useful to further characterize the efOA in vitro model.

Vacuole-like structures were observed in efOA-CPs in our study (Figure 2 and Figure 3). We confirmed that the number and size of vacuole-like structures were both significantly increased in efOA-CPs (Figure S3). Furthermore, we assumed that the increasing size of the vacuoles within the efOA pellets might affect the increasing pellet size, rather than the increase of vacuole numbers. At first, we simply hypothesized that it might be associated with chondrocyte hypertrophy which is a process that affects OA progression. We confirmed the expression of hypertrophic markers such as *COL1A1*, *COL10A1*, *RUNX2*, *MMP13*, and *VEGFA*; yet remains unclear as to what the vacuole-like structures might actually represent. Vacuole-like structures with visible vacuolar degeneration was also reported by Zhang et al. in end-stage OA patient derived cartilage tissues [40]. The authors analyzed the relationship between discoidin domain-containing receptor 2 (DDR2) and the hypertrophy caused by the terminal differentiation of chondrocytes in OA pathogenesis. DDR2 and collagen type II were upregulated in early-stage OA cartilage samples; however, the expression levels were significantly reduced in progressed late-stage OA. The authors concluded that the upregulation of DDR2 during the early stages of OA might be a response to cartilage damage and chondrocyte apoptosis, suggesting DDR2 might trigger the increased expression of hypertrophic markers. Based on this previous study, confirming the DDR2 expression in our early-stage efOA-CP models is essential. The analysis of additional time points may be useful to better understanding the characteristics of efOA and the vacuole-like structures. Knockdown experiments using siRNAs against IL-6, MMP1, and MMP10 may provide clues regarding what these vacuole-like structures represent.

We confirmed the characteristics of the generated efOA-CPs on days 7 and 21 of differentiation. While several OA-related characteristics were confirmed in the efOA-CPs, we observed that the results of disease modeling may depend on the time point of analysis. Day 7 CPs were selected for analysis, since it was the time point at which the CPs of the two groups started to show significant differences in size and morphology. These results suggested that the time point of analysis might be critical in disease modeling using patient-derived hiPSCs. Therefore, standardization of the time point of analysis must be considered when modeling certain diseases using hiPSCs in future.

## 5. Conclusions

In conclusion, we successfully generated iPSCs from fibroblasts of patients with efOA. The characteristics of early-stage day 7 CPs differed significantly between the two groups and elevated levels of IL-6, MMP1, and MMP10 were observed in efOA-CPs. The early-stage efOA-CPs showed low *COL2A1* expression and elevated expression of hypertrophic markers. Taken together, we present proof-of-concept regarding the possibility of efOA modeling using patient-derived iPSCs and suggest a possible pathological candidate that may contribute to cartilage degeneration.

## Figures and Tables

**Table 1:** Primers used for real time polymerase chain reaction (PCR) against pluripotent markers and chondrogenic markers.

Description	Target Name	REFSEQ_ID	Direction	Primer Sequence (5′–3′)	Size
Pluripotency marker	*OCT4*	NM_203289.5	Forward	GGGAAATGGGAGGGGTGCAAAAGAGG	151
			Reverse	TTGCGTGAGTGTGGATGGGATTGGTG	
	*KLF4*	NM_004235.4	Forward	TTCCCATCTCAAGGCACAC	158
			Reverse	GGTCGCATTTTTGGCACT	
	*NANOG*	NM_024865.2	Forward	AAAGGCAAACAACCCACT	270
			Reverse	GCTATTCTTCGGCCAGTT	
	*LIN28*	NM_024674.4	Forward	GTTCGGCTTCCTGTCCAT	122
			Reverse	CTGCCTCACCCTCCTTCA	
Chondrogenic marker	*SOX9*	NM_000346	Forward	GACTTCCGCGACGTGGAC	99
			Reverse	GTTGGGCGGCAGGTACTG	
	*COL2A1*	NM_001844	Forward	GGCAATAGCAGGTTCACGTACA	79
			Reverse	CGATAACAGTCTTGCCCCACTTA	
	*COL1A1*	NM_000088.3	Forward	TCTGCGACAACGGCAAGGTG	146
			Reverse	GACGCCGGTGGTTTCTTGGT	
Hypertrophy marker	*COL10A1*	NM_000493.3	Forward	CAGGCATAAAAGGCCCAC	108
			Reverse	GTGGACCAGGAGTACCTTGC	
	*RUNX2*	NM_001024630	Forward	CCAGATGGGACTGTGGTTACTG	65
			Reverse	TTCCGGAGCTCAGCAGAATAA	
	*VEGFA*	NM_003376.6	Forward	CTACCTCCACCATGCCAAGT	109
			Reverse	GCAGTAGCTGCGCTGATAGA	
	*MMP13*	NM_002427.4	Forward	TCCCAGGAATTGGTGATAAAGTAGA	123
			Reverse	CTGGCATGACGCGAACAATA	
Inflammatory cytokines	*IL1B*	NM_000576.2	Forward	ACAGATGAAGTGCTCCTTCCA	73
			Reverse	GTCGGAGATTCGTAGCTGGAT	
	*IL6*	NM_000600.5	Forward	GGTACATCCTCGACGGCATCT	81
			Reverse	GTGCCTCTTTGCTGCTTTCAC	
	*TNFA*	NM_000594.4	Forward	CTTCTCCTTCCTGATCGTGG	266
			Reverse	GCTGGTTATCTCTCAGCTCCA	
MMPs	*MMP1*	NM_002421.4	Forward	CTGGCCACAACTGCCAAATG	103
			Reverse	CTGTCCCTGAACAGCCCAGTACTTA	
	*MMP10*	NM_002425.3	Forward	CATTCCTTGTGCTGTTGTGTC	225
			Reverse	TGTCTAGCTTCCCTGTCACC	
House-keeping gene	*GAPDH*	NM_002046.5	Forward	ACCCACTCCTCCACCTTTGA	101
			Reverse	CTGTTGCTGTAGCCAAATTCGT	

## Data Availability

Data is contained within the article or supplementary material.

## Supplementary Materials

The supplementary materials are available online at https://www.mdpi.com/2073-4409/10/2/317/s1.
