# Supplementary material for: Characterization of Early-Onset Finger Osteoarthritis-Like Condition Using Patient-Derived Induced Pluripotent Stem Cells
Source: Cells. 2021 Feb 4;10(2):317. doi: 10.3390/cells10020317 (PMC7913990; doi:10.3390/cells10020317)
Supplement: Supplementary file 1 [file cells-10-00317-s001.pdf]

Supplementary Figures

- *Journal*: Cells
- *Manuscript #*: cells-1018009
- *Title of Paper*: Characterization of early-onset finger osteoarthritis-like condition using patient-derived induced pluripotent stem cells
- *Authors*: Yeri Alice Rim (First author), Yoojun Nam, Narae Park, Kijun Lee, Hyerin Jung, Seung Min Jung, Jennifer Lee, and Ji Hyeon Ju (Corresponding author)*

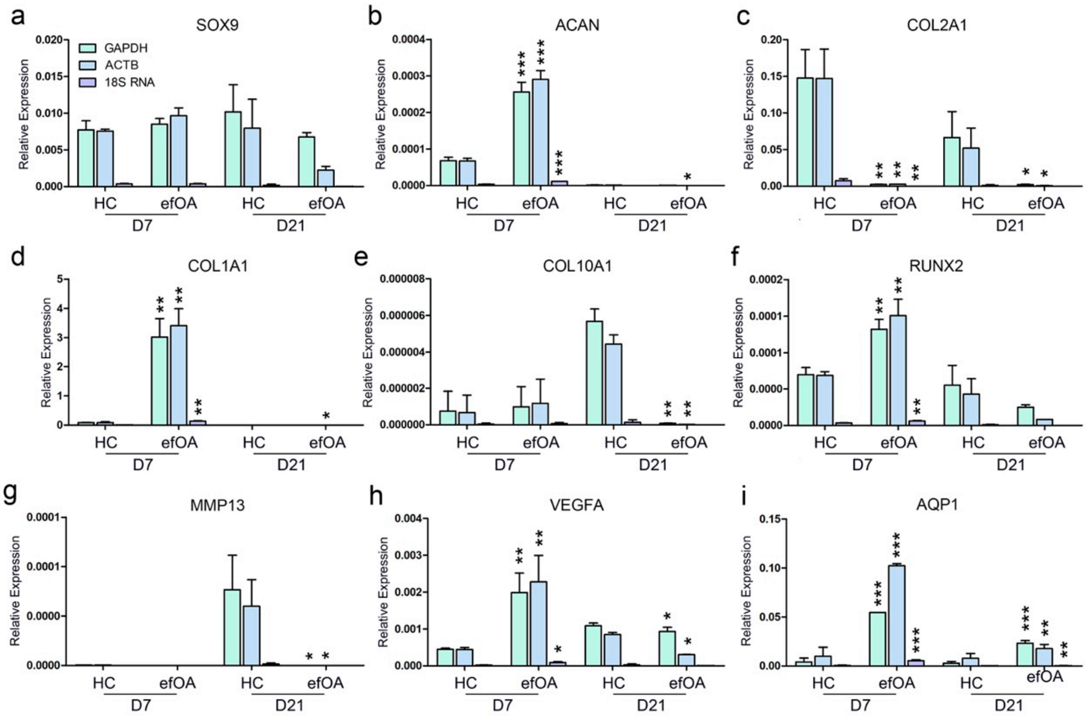

Figure S1. Gene expression confirmation using three different reference genes; *GAPDH*, *β-actin (ACTB)*, and *18S RNA*. (**a**) Relative expression of *SOX9*. (**b**) Relative expression of *ACAN*. (**c**) Relative expression of *COL2A1*. (**d**) Relative expression of *COL1A1*. (**e**) Relative expression of *COL10A1*. (**f**) Relative expression of *RUNX2*. (**g**) Relative expression of *MMP13*. (**h**) Relative expression of *VEGFA*. (**i**) Relative expression of *AQP1*. Significant differences between the healthy control (HC) and early finger osteoarthritis (efOA) on each time point (day 7 and 21; D7 and D21) are each indicated by the asterisk symbol (* $p < 0.05$, ** $p < 0.01$, *** $p < 0.001$).

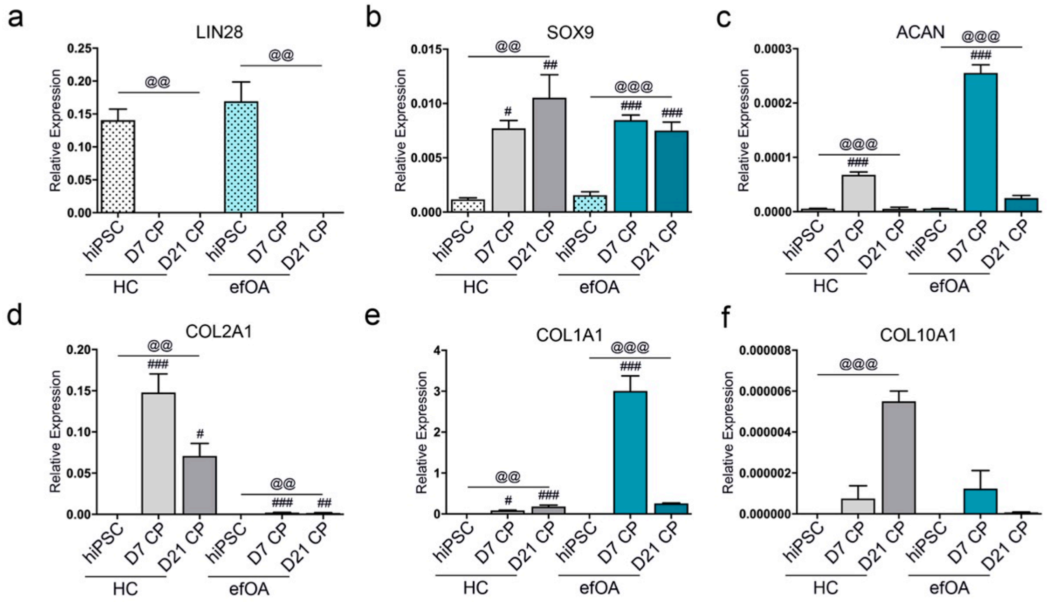

Figure S2. Confirmation of chondrogenic gene expression in human induced pluripotent stem cells (hiPSCs) and derived chondrogenic pellets (CPs). (**a**) Relative expression of *LIN28*. (**b**) Relative expression of *SOX9*. (**c**) Relative expression of *ACAN*. (**d**) Relative expression of *COL2A1*. (**e**) Relative expression of *COL1A1*. (**f**) Relative expression of *COL10A1*. Statistically significant differences between the hiPSC control and CPs on each time point are indicated by the hash symbol (# $p < 0.05$, ## $p < 0.01$, ### $p < 0.001$). The significance between each group analyzed using ANOVA was indicated using the at sign (@ $p < 0.05$, @@ $p < 0.01$, @@@ $p < 0.001$).

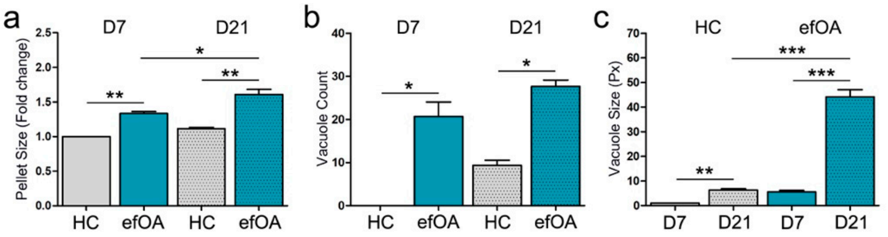

Figure S3. Further characterization of day 7 (D7) and 21 (D21) chondrogenic pellets (CPs). (**a**) Pellet size measurement of CPs. (**b**) Vacuole count in CPs. Statistical significance between healthy control (HC) and early finger osteoarthritis (efOA) CPs are indicated by the asterisk symbol (* $p < 0.05$, ** $p < 0.01$, *** $p < 0.001$). (**c**) Measurement of vacuole size in CPs. The statistical significance was measured between D7 HC-CPs and other groups. The expression of each gene was normalized to that of GAPDH.

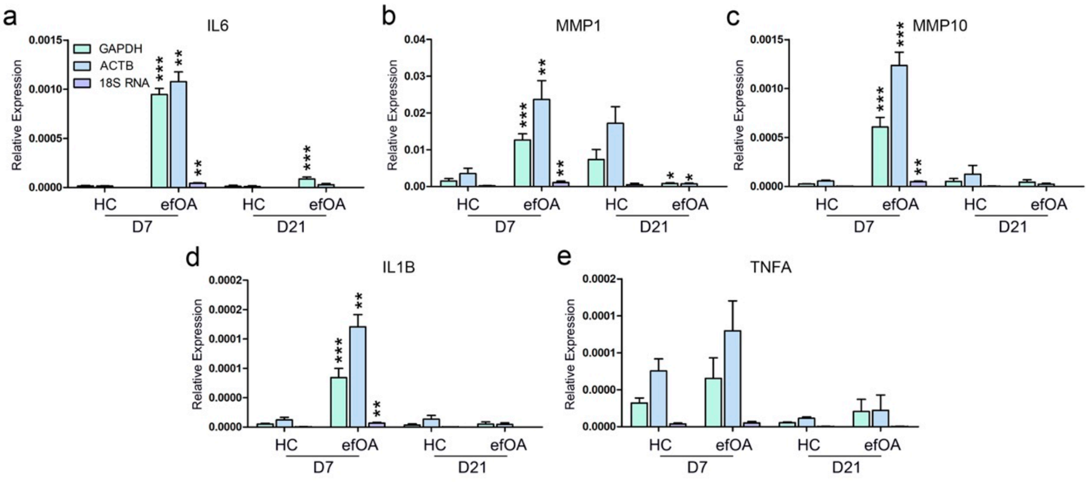

Figure S4. Gene expression confirmation using three different reference genes; *GAPDH*, *β-actin (ACTB)*, and *18S RNA*. (**a**) Relative expression of *IL6*. (**b**) Relative expression of *MMP1*. (**c**) Relative expression of *MMP10*. (**d**) Relative expression of *IL1B*. (**e**) Relative expression of *TNFA*. Statistical significance between the healthy control (HC) and early finger

osteoarthritis (efOA) on each time point (day 7 and 21; D7 and D21) are each indicated by the asterisk symbol (* $p < 0.05$, ** $p < 0.01$, *** $p < 0.001$).

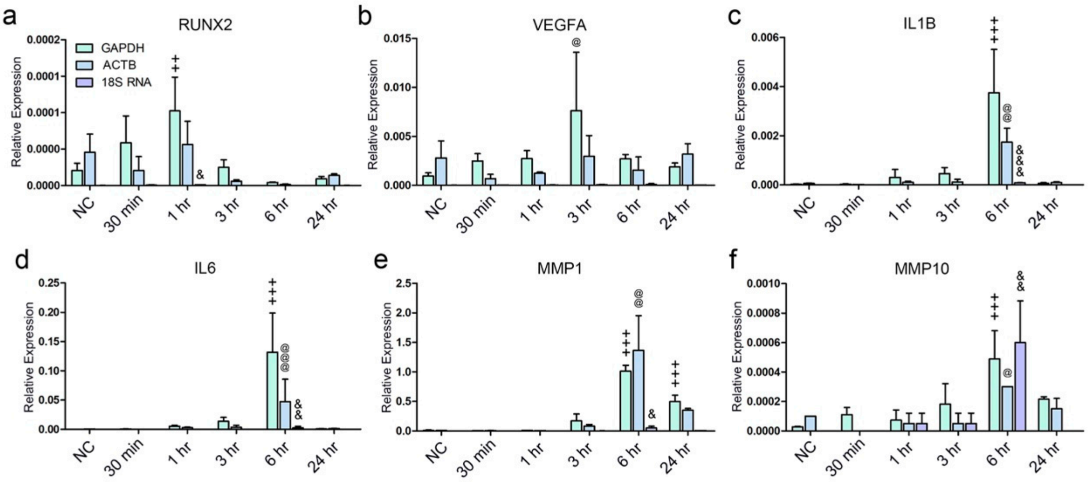

Figure S5. Confirmation of *RUNX2*, *VEGFA*, *IL1B*, *IL6*, *MMP1*, and *MMP10* expression using three different reference genes; *GAPDH*, *β-actin (ACTB)*, and *18S RNA*. (**a**) Relative expression of *RUNX2*. (**b**) Relative expression of *VEGFA*. (**c**) Relative expression of *IL1B*. (**d**) Relative expression of *IL6*. (**e**) Relative expression of *MMP1*. (**f**) Relative expression of *MMP10*. Statistically significant differences between the normal control (NC) and the treatment groups are each indicated by the +, @, and & (One-way ANOVA, Dunnett's test, +,@,& $p < 0.05$, ++,@@,&& $p < 0.01$, +++,@@@,&&& $p < 0.001$).
